# Validation of the Brazilian Version of the World Health Organization Disability Assessment Schedule (WHODAS 2.0) for Women After Breast Cancer Surgery

**DOI:** 10.1002/pri.70106

**Published:** 2025-09-16

**Authors:** Lia Rodrigues Rocha, Thalia Oliveira Ximenes, Elidia Nascimento da Silva, Ana Karoline da Silva de Araújo, Jennifer Rego Pereira, Simony Lira Nascimento

**Affiliations:** ^1^ Master Program in Physiotherapy and Functioning (PPGFisio) Physiotherapy Department Federal University of Ceará (UFC) Fortaleza Brazil; ^2^ Physiotherapy Department Federal University of Ceará (UFC) Fortaleza Brazil

**Keywords:** breast cancer, functioning, patient reported outcome measures, validation study

## Abstract

**Background and Purpose:**

The World Health Organization Disability Assessment Schedule (WHODAS 2.0) is a generic instrument created by the World Health Organization (WHO) for measuring functioning and disability. Although this instrument has been extensively studied in different populations, its validity concerning breast cancer patients who have undergone surgery remains uncharted. This study aims to analyze the measurement properties of the Brazilian version of WHODAS 2.0 to validate it as a tool for assessing the functioning/disability of postoperative breast cancer surgery patients.

**Methods:**

This is a validation study conducted at three centers in the northeast of Brazil, involving women between 3 and 12 months after mastectomy or quadrantectomy, interviewed with the Brazilian version of the 36‐item WHODAS 2.0. Participants also completed the Functional Assessment of Cancer Therapy (FACT‐B+4) questionnaire. Internal consistency was assessed using Cronbach's alpha. Intraclass correlation coefficients (ICC), standard error of measurement (SEM) and smallest detectable change (SDC) were used to measure test‐retest reliability. Both construct and convergent validity were analyzed using Spearman correlation.

**Results:**

Ninety‐five women with a mean age of 53.12 (± 11.10) answered the WHODAS. The responses revealed satisfactory internal consistency for all domains (*α* ≥ 0.7) and the total score (*α* = 0.93). Excellent test‐retest reliability was observed (ICC = 0.87). Regarding the construct validity, a strong negative correlation was found between FACT‐B+ 4 and WHODAS (*ρ* = −0.75). Moreover, the convergent validity analysis depicted correlations (between WHODAS and FACT‐B+ 4 domains) ranging from weak to moderate, except for the “mobility” subscale of WHODAS, which showed a strong correlation with the “physical well‐being” subscale of FACT‐B+ 4.

**Discussion:**

The results provide evidence that the WHODAS 2.0 is a valid and reliable instrument for assessing the functioning/disability of the target population, which allows its use as outcome measure based on the biopsychosocial model.

## 1 Introduction

Breast cancer is a relevant health problem as it represents the most common type of cancer in women worldwide (Bray et al. 2018). In Brazil, according to the National Cancer Institute, the estimated number of new cases of breast cancer for the triennium 2023–2025 is 73,610. This corresponds to an estimated risk of 66.54 new cases per 100,000 women. Additionally, breast cancer has a high mortality rate, causing 18,139 deaths in Brazil only in 2021 (Brazilian National Cancer Institute, 2022). Despite the increasing improvement in early breast cancer diagnosis, most cases in Brazil are still diagnosed at more advanced tumor stages, leading to the implementation of more radical treatments, such as mastectomy surgeries, followed by adjuvant therapy (Brazilian National Cancer Institute, 2019).

Surgeries, in addition to causing physical‐functional alterations such as a decrease in shoulder range of motion, also result in other adverse effects that typically negatively impact the quality of life. For instance, surgeries can lead to a deterioration of psychological aspects, including depression, anxiety, low self‐esteem, and distortion of body image. These changes contribute to an increase in morbidity and mortality among women (Moro‐Valdezate et al. 2013).

According to the International Classification of Functioning, Disability and Health (ICF), functioning is a broad concept resulting from the interaction of components such as health conditions, body function and structure, activity, participation, and personal and environmental factors. Functioning assessment is crucial since it allows the identification of impairments in these components. Moreover, early functioning assessment enables preventive strategies to avoid the worsening or onset of more severe disabilities (Ferrer et al. 2019).

Based on the theoretical‐conceptual framework of the ICF, the World Health Organization (WHO) developed the World Health Organization Disability Assessment Schedule (WHODAS 2.0) (Ustun et al. 2010)—a standardized tool for assessing health and disability across different cultures. This instrument evaluates the level of functioning in six life domains: cognition, mobility, self‐care, interpersonal relationships, activities of daily living, and participation in society and provides a quantitative outcome for each domain and total score, and facilitates its use in clinical practice (World Health Organization 2013; Castro and Leite 2017; Silva et al. 2013).

The measurement properties of the WHODAS 2.0 have been studied in various cultures and different health conditions. For instance, Zhao et al. (Zhao et al. 2013) validated the Chinese version for breast cancer patients undergoing chemotherapy. The use of validated questionnaires is essential to reduce the risk of bias or incorrect results. In other words, failures in the measurement/assessment process can lead to incorrect conclusions about the outcomes of interventions in clinical studies and/or provide biased estimates of patients' functioning (Zhao et al. 2013; Jamshidi et al. 2022). In addition, it is worth noting that there is a general lack of questionnaires that encompass the domains of the biopsychosocial model. Therefore, validation studies of the WHODAS 2.0 for various populations become even more relevant.

Given the high prevalence of breast cancer and the fact that the measurement capabilities of the WHODAS 2.0 have not been tested for Brazilian patients undergoing breast cancer surgeries, this study aims to analyze the measurement properties (internal consistency, reproducibility, construct validity, and convergent validity) of the WHODAS 2.0 to validate it as a tool for assessing the functioning/disability of this population. We also provide reliability measures based on standard error of measurement (SEM) and the smallest detectable change (SDC) to contribute to the clinical meaningfulness of the WHODAS 2.0 scores to clinicians and researchers.

## 2 Methods

### 2.1 Study Design

This work constitutes a validation study (cross‐sectional) of the Brazilian version of the 36‐item WHODAS 2.0 for women in the postoperative period of breast cancer surgery. The present study followed the guidelines from Strengthening the Reporting of Observational Studies in Epidemiology (STROBE).

### 2.2 Participants

The participants included women aged between 18 and 80 years who underwent quadrantectomy or mastectomy surgeries (within 3–12 months post‐surgery). This study considered unilateral or bilateral surgeries, with or without breast reconstruction, and with or without an axillary approach (lymphadenectomy or sentinel lymph node biopsy) as a form of breast cancer treatment. As exclusion criteria, we adopted: (i) women with possible cognitive impairments unable to respond to self‐report questionnaires, (ii) women who had been hospitalized in the last 30 days, and (iii) women with indications for sectoral resection or removal of nodules. Regarding the sample size, we followed guidelines from the Consensus‐based Standards Measurements Instruments (COSMIN) checklist, which deems a sample size above 50 participants appropriate for assessing the measurement properties of questionnaires (Gagnier et al. 2021; L. B. Mokkink et al. 2019).

### 2.3 Instruments

This study employed the following instruments for data collection: (i) Mini‐Mental State Examination (MMSE) (Brucki et al. 2003), (ii) Brazilian version of the WHODAS 2.0 (Ustun et al. 2010; Castro and Leite 2017; Castro et al. 2015), (iii) Functional Assessment of Cancer Therapy—Lymphedema (FACT‐B+ 4) (Coster et al. 2001), and (iv) a general standardized form related to sociodemographic and clinical data.

The MMSE was used to screen for cognitive impairments. Since this was a validation study, we tested the candidates' ability to answer the questionnaires. The threshold acceptance score relies on the candidates' level of education: 20 points for illiterates, 25 for those with 1–4 years of education, 26.5 for those with 5–8 years, 28 for those with 9–11 years, and 29 for candidates with more than 11 years (Brucki et al. 2003).

WHODAS 2.0 is a generic patient‐reported outcome measure (PROM) for disability and functioning assessment. Its translation to Portuguese and adaptation for Brazil was developed through a formal partnership with the WHO (Castro and Leite 2017; Castro et al. 2015). The instrument consists of multiple‐choice questions that assess the level of functioning in six domains of life: cognition (D1, 6 questions), mobility (D2, 5 questions), self‐care (D3, 4 questions), getting along with people (D4, 5 questions), life activities (D5, 4 or 8 questions depending on the occupation status) and participation in society (D6, 8 questions). WHODAS 2.0 provides scores for each of the six domains and an overall (total) score, which can range from 0 to 100, where 0 means no disability and 100 means complete disability. To handle missing data, we followed official guidelines: if one or two answers (items) are missing in a given domain, the average answer computed using the available items within that domain is assigned as a response to the missing answers (Ustun et al. 2010; Castro and Leite 2017). Patients who were not involved in work activities answered the 32‐item version of the WHODAS.

FACT‐B+ 4 is a valid and widely used instrument for measuring the quality of life in breast cancer patients (Oliveira et al. 2014). This instrument contains questions regarding general aspects of quality of life, as well as specific issues related to breast cancer and arm morbidity. It consists of 41 questions and is divided into six domains: physical well‐being (PWB), emotional well‐being (EWB), functional well‐being (FWB), social/family well‐being (SWB), Breast Cancer subscale (BCS), and Lymphedema subscale (LS). Moreover, FACT‐B+ 4 provides scores separately for each scale, which are added to obtain the total score—ranging from 0 to 164. The higher the score, the better the patient's quality of life. For missing data, we used the average of the within‐domain answered questions.

Sociodemographic variables included age, marital status, school attendance, monthly family income and skin color. Regarding clinical/surgical information, we collected body mass index, diabetes (yes/no), hypertension (yes/no), adjuvant therapy (yes/no), cancer stage (I–IV), physiotherapy participation (yes/no), operated breast (left, right, bilateral), surgery type (quadrantectomy/mastectomy), axillary approach (Lymphadenectomy, sentinel lymph node, no information), and breast reconstruction (yes/no).

### 2.4 Data Collection

The study was conducted at three centers: the Assis Chateaubriand School Maternity, the Integrated Oncology Center, and the Group of Education and Oncological Studies—all located in Fortaleza/Brazil. The data were collected from March 2022 to August 2023. The Ethics committee approved the project. The selection process was convenience based, involving the invitation of patients undergoing follow‐up at the Mastology outpatient clinics of the institutions through open demand. Patients were invited to participate in the research, and signing the Informed Consent Form was requested for those who accepted. Subsequently, they completed the Mini‐Mental State Examination. Then, eligible patients filled out a form with sociodemographic and clinical data. Finally, the WHODAS and FACT‐B+ 4 instruments were applied through face‐to‐face interviews. In the WHODAS retest phase, a researcher contacted the patient by phone 7–14 days after the first interview.

To ensure consistency during data collection, five researchers were selected and received specific training for applying the WHODAS. In particular, we offered a 40‐h course via the virtual environment of the NUTEDS—*Núcleo de Tecnologias e Educação a Distância em Saúde (*Center for Technologies and Distance Education in Health) at the Federal University of Ceará. We utilized the Brazilian version of the WHODAS manual, which includes the standardized questionnaire in Brazilian Portuguese available at (https://iris.who.int/bitstream/handle/10665/43974/9788562599514_por.pdf) (Castro et al. 2015). In addition, all researchers received detailed instructions regarding approaching potential participants, completing the sociodemographic and clinical data form, and applying FACT‐B+ 4.

### 2.5 Statistical Analysis

Python was the software tool used for statistical data analysis. Sample characterization was carried out through descriptive statistics (frequency)—continuous variables were stratified to ease interpretation. We employ the complex scoring mechanism of the WHODAS 2.0, following the instrument's manual (Ustun et al. 2010; Castro et al. 2015). We report statistics (max, min, mean, and standard deviation) of WHODAS scores for each subscale and the total score. In addition, we computed Pearson correlation coefficients (denoted by *r*) between each domain‐level score and the total one. Regarding internal consistency, we consider Cronbach's alpha (*α*) coefficients and interpret values between 0.7 and 0.95 as satisfactory consistency (McKinley et al. 1997). The test‐retest reliability of the WHODAS 2.0 was examined using intraclass correlation coefficients (ICC) from a two‐way mixed‐effects model (single measure and absolute agreement). To interpret the obtained results, we adopt the following standard: values below 0.5 (poor), between 0.5 and 0.75 (moderate), between 0.75 and 0.9 (good), and above 0.9 (excellent) (Koo and Li 2016).

To obtain the SEM and SDC values, we first computed the differences between the test and retest WHODAS scores, that is, we created a variable called difference, defined as difference = test_score − restest_score. Next, we calculated the standard deviation of this variable and denoted it by SD_difference._ Finally, we computed the SEM and SDC values using the following formulas: $\text{SEM}={\text{SD}}_{\text{difference}}*\sqrt{1-\text{ICC}}$ and $\text{SDC}=1.96*\sqrt{2*\text{SEM}}$ (Polit 2014).

To construct validity analysis, Spearman correlation coefficients (denoted by *ρ*) were calculated to measure correlations between the total scores of WHODAS 2.0 and FACT‐B+ 4. For completeness, statistics (min, max, mean, standard deviation) for the obtained FACT‐B+ 4 scores are also reported. Furthermore, convergent validity was assessed through pairwise (Spearman) correlation analysis between the subscale/total scores of WHODAS 2.0 and FACT‐B+ 4. Correlation values are interpreted as follows: 0.9 or above (in absolute value) indicates a very strong correlation; 0.7 to 0.9 denotes a strong correlation; 0.5 to 0.7 means a moderate correlation; and 0.3 to 0.5 denotes a weak correlation (Mukaka 2012). The significance level was established at 5% for all analyses.

## 3 Results

Initially, 133 women were recruited for the study. However, 38 women were excluded from not reaching the acceptance threshold score in the Mini‐Mental State Examination. Consequently, our sample consisted of 95 participants, with 75 of them answering both interviews (i.e., test and retest).

An overview of the sociodemographic/clinical/surgical data of participants is provided in Table 1. Most participants were between 40 and 59 years old, married, self‐declared as mixed‐race, had a low level of education (1–8 years of study) and low income (up to 1 minimum wage). Regarding the clinical status, 37 (39%) had chronic hypertension and 16 (17%) had diabetes. Additionally, 59 (62%) had a family history of cancer, and 37 (39%) were overweight. Concerning staging, the most common type was Stage II, with 43 patients (46%). Regarding adjuvant treatment, 76 (80%) underwent radiotherapy, 42 (44%) received hormonotherapy, and 51 (53%) underwent chemotherapy. The surgical characteristics of the sample revealed that 46 (49%) had surgery on the left breast, 49 (51%) had undergone quadrantectomy, 32 (34%) had a sentinel lymph node biopsy, and only 24 (25%) underwent breast reconstruction. In terms of rehabilitation, 46 (49%) underwent or were undergoing physiotherapeutic treatment.

**TABLE 1:** Description of the sociodemographic, clinical and surgical variables associated with women after breast cancer surgery who answered the WHODAS (*n* = 95).

Type	Variable	Values	Statistics *N* (%)
Sociodemographic	Age (years)	≤ 40	10 (11)
		> 40 & ≤ 60	57 (60)
		> 60	28 (29)
	Marital status	Single	28 (29)
		Married	49 (52)
		Divorced	11 (12)
		Widowed	7 (7)
	Skin color	White	29 (31)
		Black	6 (6)
		Mixed‐race	59 (62)
		Yellow	1 (1)
	School attendance	Less than 8 years	38 (40)
		Between 8 and 11 years	33 (35)
		More than 11 years	23 (24)
		NA	1 (1)
	Monthly family income	≤ 1 minimum wage	38 (40)
		> 1, ≤ 2 minimum wages	23 (24)
		> 2 minimum wages	34 (36)
Clinical	Hypertension	Yes	37 (39)
		No	58 (61)
	Diabetes	Yes	16 (17)
		No	79 (83)
	Family history of cancer	Yes	59 (62)
		No	36 (38)
	Body mass index (BMI)	> 18.5 & ≤ 25 (healthy weight)	27 (29)
		> 25 & ≤ 30 (overweight)	37 (39)
		> 30 (obesity)	23 (24)
		NA	8 (8)
	Adjuvant chemotherapy	Yes	51 (53)
		No	44 (47)
	Adjuvant hormonotherapy	Yes	42 (44)
		No	53 (56)
	Adjuvant radiotherapy	Yes	76 (80)
		No	19 (20)
	Tumor staging	Type I	5 (5)
		Type II	43 (46)
		Type III	21 (22)
		Type IV	6 (6)
		NA	20 (21)
	Physiotherapy	Yes	46 (49)
		No	49 (51)
Surgical	Operated breast	Right	43 (45)
		Left	46 (49)
		Bilateral	6 (6)
	Surgery type	Quadrantectomy	49 (51)
		Mastectomy	46 (49)
	Axillary approach	Lymphadenectomy	31 (32)
		Sentinel lymph node biopsy	31 (34)
		No information	32 (34)
	Breast reconstruction	Yes	24 (25)
		No	71 (75)

Abbreviation: NA, no answer.

Table 2 reports the maximum, minimum, mean, and standard deviation (SD) values of the total and domain scores of WHODAS 2.0. The average total score was 24.64. The domains with the highest scores were “Life Activities” (33.51 ± 28.32) and “Participation” (32.36 ± 21.70). On the other hand, the lowest scores were observed in “Self‐care” (13.06 ± 17.69) and “Getting along with people” (14.88 ± 17.59). The best linear predictors of the total score were “Cognition” and “Mobility” with coefficients equal to 0.84 and 0.85, respectively. Except for the domain “Getting along with people”, all correlation values are good.

**TABLE 2:** Descriptive statistics, internal consistency via Cronbach's *α*, and test‐retest reliability, that is, intraclass correlation (ICC) scores and 95% confidence intervals of WHODAS scores obtained from women undergoing surgery for breast cancer.

Domain	Min—max	Mean ± SD	*r* ^a^	Cronbach's *α*	ICC [95% CI]^b^
Cognition	0.00–85.00	22.60 ± 20.47	0.84	0.83	0.67 [0.52 0.78]
Mobility	0.00–87.50	23.79 ± 23.77	0.85	0.82	0.68 [0.53 0.78]
Self‐care	0.00–80.00	13.06 ± 17.69	0.75	0.63	0.59 [0.42 0.72]
Getting along	0.00–75.00	14.88 ± 17.59	0.63	0.67	0.77 [0.66 0.85]
Life activities	0.00–100.00	33.51 ± 28.32	0.80	0.89	0.75 [0.62 0.83]
Participation	0.00–87.50	32.36 ± 21.70	0.80	0.83	0.84 [0.76 0.90]
Total score	0.00–69.57	24.64 ± 17.16	1.00	0.93	0.87 [0.80 0.91]

Abbreviations: Max, maximum, Min, minimum; SD, standard deviation.

^a^ Pearson correlation test between each domain and the total score.

^b^ All results are statistically significant (i.e., *p*‐value < 0.05).

In addition, Table 2 depicts the reliability results. Regarding internal consistency, in five out of 7 domains, we observed satisfactory consistency (*α* ≥ 0.7). Importantly, the Cronbach's alpha for the total WHODAS score was 0.93. On the other hand, the smallest value was achieved for the domain “Self‐care” (*α* = 0.63).

**FIGURE 1:**
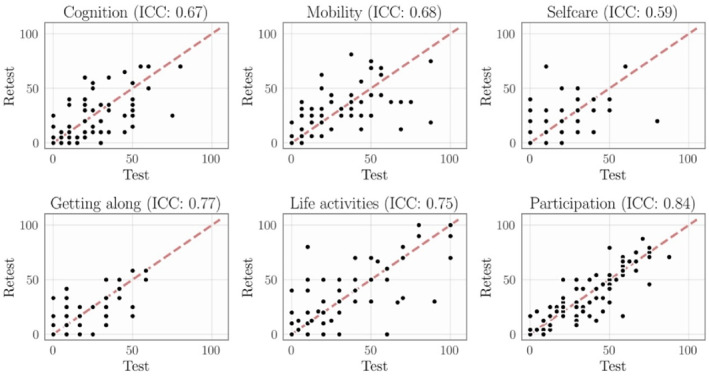
Scatter plots of the test‐retest scores for each domain of the WHODAS 2.0 (*n* = 75). Intraclass correlation coefficients (ICC) values for each domain. For reference, the red line denotes the identity map between the test and retest scores. Overall, the domain “Participation” produced the best test‐retest correlation.

To assess the test‐retest reliability, considering 75 participants due to a loss of 20 participants who did not respond to the retest, the highest ICC values were obtained for total score (0.87) and “Participation” (0.84), indicating good test‐retest reliability. The domains “Cognition”, “Mobility”, and “Getting along” achieved moderate ICC values. For a visual assessment of the test‐retest scores, Figure 1 shows scatter plots for each domain. This validates the “Participation” domain as the most reliable one. In addition, for the total WHODAS score, we observed an average difference between the test and re‐test of −0.40 (and SD = 2.32), resulting in SEM = 0.84 and SDC = 2.32 (95% CI: 0.68—3.96) points.

**FIGURE 2:**
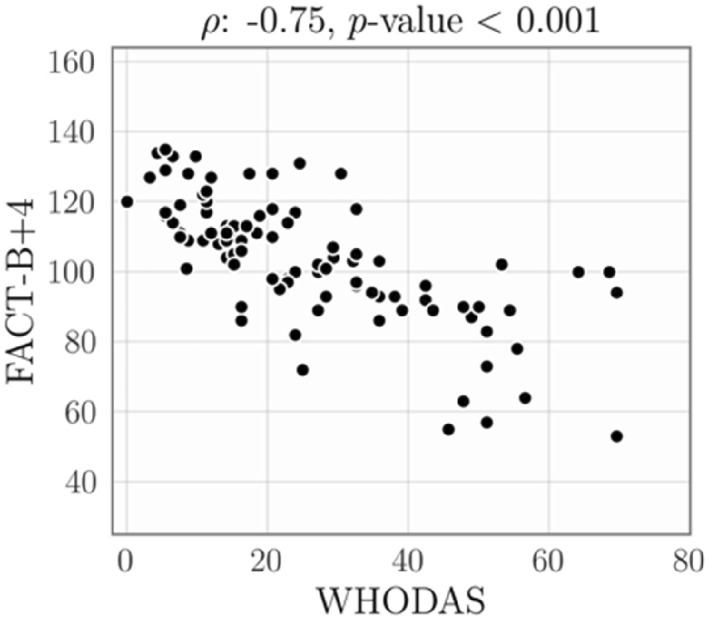
Scatter plot between FACT‐B+ 4 versus WHODAS 2.0 total scores. We observed a negative correlation (e.g., patients with high FACT‐B+ 4 scores tend to have low WHODAS scores), validated with a Spearman correlation of −0.75.

Regarding the construct validity, the Spearman correlation coefficient between the WHODAS and FACT‐B+ 4 scores is *ρ* = −0.75. The negative correlation value is because higher scores on FACT‐B+ 4 indicate better quality of life, while higher scores on WHODAS indicate greater disability. Therefore, the correlation value found represents a strong relationship between the overall scores of the two instruments. Figure 2 shows the total scores for the WHODAS and FACT‐B+ 4 instruments. We can observe a linear trend between the scores of these instruments.

Table 3 presents statistics for the values obtained using FACT‐B+ 4. Except for the social/family well‐being (*r* = 0.49) and lymphedema (*r* = 0.55) subscale domains, the scores for each domain showed good correlation with the total score, that is, values greater than 0.7.

**TABLE 3:** Statistics (minimum, maximum, mean, and standard deviation) of the FACT‐B+ 4 scores obtained from women undergoing surgery for breast cancer.

Subscale	Min—Max	Mean ± SD	*r* ^a^
Physical well‐being (PWB)	8.00–28.00	21.38 ± 4.90	0.79
Social/family well‐being (SWB)	6.00–28.00	19.48 ± 4.70	0.49
Emotional well‐being (EWB)	2.00–24.00	19.01 ± 4.08	0.74
Functional well‐being (FWB)	3.00–27.00	17.45 ± 4.87	0.78
Breast‐cancer subscale (BCS)	6.00–37.00	26.63 ± 6.50	0.75
Lymphedema subscale (LS)	0.00–20.00	15.67 ± 4.20	0.55
Total score	53.00–135.00	103.96 ± 17.88	1.00

^a^ Pearson correlation between each subscale and the total score.

For assessing the convergent validity, Table 4 shows the Spearman correlation coefficients between the domains of WHODAS 2.0 and FACT‐B+ 4 along with their respective levels of statistical significance. The domains PWB (Physical well‐being) and LS (Lymphedema subscale) obtained moderate correlation with almost all WHODAS domains except for the “mobility” domain of WHODAS, which showed a strong correlation with the “physical well‐being” domain of FACT‐B+ 4 (*ρ* = −0.73).

**TABLE 4:** Pairwise Spearman correlations (*ρ*) between the domains of WHODAS 2.0 and FACT‐B+4 scores obtained from women undergoing surgery for breast cancer.

	Cognition	Mobility	Self‐care	Getting along	Life activities	Participation
PWB	0.68*	−0.73*	−0.50*	−0.44*	−0.62*	−0.56*
SWB	−0.10 (0.320)	−0.14 (0.192)	−0.13 (0.212)	−0.26*	−0.04 (0.719)	0.02 (0.854)
EWB	−0.27*	−0.34*	−0.34*	−0.30*	−0.36*	−0.45*
FWB	−0.29*	−0.45*	−0.34*	−0.26*	−0.40*	−0.50*
BCS	−0.39*	−0.44*	−0.36*	−0.42*	−0.43*	−0.55*
LS	−0.56*	−0.55*	−0.51*	−0.27*	−0.56*	−0.53*

*Note:* We use * to denote statistically significant results (i.e., *p*‐value ≤ 0.05). For non‐significant results, we report *p‐*values within parenthesis.

Abbreviations: BCS: Breast cancer subscale; EWB: Emotional well‐being, FWB: Functional well‐being; LS: Lymphedema subscale; PWB: Physical well‐being, SWB: Social/family well‐being.

## 4 Discussion

This study evaluated several measurement properties of the Brazilian version of the 36‐item WHODAS instrument. We tested the following properties: internal consistency, test‐retest reliability, construct validity, and convergent validity, with the goal to validate the WHODAS 2.0 as a tool to measure functioning/disability in postoperative breast cancer surgery patients.

Overall, our findings revealed satisfactory internal consistency for both domain and total scores (Cronbach's alphas greater than 0.7). For the domains “self‐care” and “getting along”, we observed moderate values (i.e., 0.6 < *α* < 0.7). It is well‐known that Cronbach's *α* values tend to increase with the number of items (Tavakol and Dennick 2011). Therefore, a possible explanation for the lower value for the domain “self‐care” is that it is the domain with the smallest number of items (4 questions). Moreover, the *α* values for “getting along” increased from 0.67 to 0.7 by simply removing the question about sexual activity (D4.5), which 32% of the participants did not answer. This high number is possibly due to discomfort in responding to something intimate and personal that is still taboo for many women. This finding aligns with other studies that reported a high proportion of missing data related to this item, such as Ovando et al. (Ovando et al. 2024), where there was a 41% missing data rate for this item. This further highlights the importance of this study for the Brazilian cancer population, where talking about sexual activity can be taboo. Furthermore, in the first year after surgery, many women are receiving adjuvant therapies and are not sexually active. After treatment, there are various physical and emotional changes that influence the sexuality of patients. According to Mendoza et al. (Mendoza et al. 2017), sexual dysfunction in breast cancer patients is common (occurring in 25%–66% of cases). The main problems include a decrease in sexual interest (49.3%), dyspareunia (35%–38%), concerns about body image (10%–14%), arousal (5%), and orgasm (5%).

Notably, our internal consistency results resemble those of similar studies. An example is the work by Lourenço et al. (Lourenço et al. 2020), who conducted a pilot study applying WHODAS 2.0 to 32 women with breast cancer to assess disability obtained *α* = 0.87 (in the present study, *α* = 0.93 was found). Other similar findings include: (i) the mean and standard deviation of total scores (27.07 ± 13.83 vs. 24.64 ± 17.16), (ii) the domains “life activities” and “participation” having higher scores, indicating a higher level of disability, and the domains “self‐care” and “getting along” having lower scores. These consistent results suggest that the Brazilian version of WHODAS is a reliable instrument for assessing the functioning and disability of postoperative breast cancer surgery patients. The moderate alpha values in specific domains highlight areas that may require further investigation or consideration in the adaptation of the instrument.

These items related to work or school activities (D5.5–D5.8) in the “life activities” domain also had a high rate of missing answers: 93%, as this study was conducted in the first year after surgery, most women were away from work to devote time to surgery recovery or adjuvant therapies. A study conducted in Germany (Schmidt et al. 2019) with patients evaluated 1 year after breast cancer surgery showed that almost half of the survivors who worked before surgery had to reduce their working hours or stop working completely before reaching retirement age. Symptoms such as fatigue, depression, memory or attention problems, arm morbidity, as well as age, educational level, and family situation have a significant impact on their lives, thus hindering their return to work. Similarly, Dumas et al. (Dumas et al. 2019) showed that after 2 years from diagnosis, 21% of the women did not return to work. Thus, it is essential that treatment protocols also include initiatives aimed at reintegration into the professional environment, as well as physical and psychosocial support.

The test‐retest reliability results (ICC = 0.87) indicate that the WHODAS 2.0 is an instrument capable of consistently reproducing results at different times (Koo and Li 2016; L. Mokkink et al. 2010). A clear linear trend can be observed in the qualitative assessment from Figure 1, particularly for the “participation” domain. The ICF defines participation as “involvement in life situations”, which is an important outcome for rehabilitation (Ustun et al. 2010).

We note that All retest absences were due to the impossibility of contacting the patients through phone calls—either they didn't answer the calls or did not have phone numbers. In the latter, patients provided relatives' phone numbers, but some were unavailable during the retest call. We also note that many participants are from the countryside. Therefore, it is hard to conclude whether the instrument played a role in the low retest attendance.

In addition, we found SEM = 0.84 and SDC = 2.32 for the WHODAS scores. These results provide central information on the reliability of the instrument by demonstrating the range in which the theoretical “true” score lies (SEM) and offer context when interpreting data from longitudinal measurements by indicating how much the score needs to change before one can reasonably be sure that an actual change has occurred (SDC). Clinicians and researchers could use the SEM and SDC values as benchmarks when interpreting WHODAS scores, both in clinical practice and as part of research projects.

In terms of construct validity, when comparing the total WHODAS scores with those from FACT‐B+ 4, a strong negative Spearman correlation of −0.75 was found. This demonstrates that WHODAS can capture the aspect of quality of life evaluated by FACT‐B+ 4, which has a similar construct. Regarding the analysis of convergent validity, it was observed that the correlations ranged from weak to moderate, except for the “mobility” subscale of WHODAS with the “physical well‐being” subscale of FACT‐B+ 4, where the correlation was strong (−0.73). These findings are similar to the study by Zhao et al. (Zhao et al. 2013), which applied WHODAS to 402 breast cancer patients receiving chemotherapy and correlated it with FACT‐B. As a result, weak to moderate correlations were obtained both in total scores and between the domains of the instruments.

Functioning has been widely studied, and various instruments to assess functional capacity have been tested, but until now, none have covered all aspects of the biopsychosocial model (Castro and Leite 2017). WHODAS 2.0 currently stands out as a consistent instrument aimed at analyzing the functioning and disability status of patients, as advocated by WHO. The questionnaire items are based on the domains of the ICF and seek an interaction of contextual factors of the patient with their health condition. In clinical practice, WHODAS provides a multifactorial view, allowing healthcare professionals to perform more specific interventions and subsequently monitor the results of these interventions. This study becomes even more important because of the high incidence rate of breast cancer in Brazil and the increased disease‐free survival (and survival with disabilities) rates achieved with improvements in early diagnosis and treatments for cancer. Another important point is that this study provides the opportunity to use functioning as a health indicator for this population as recommended by WHO.

As a limitation, it is noteworthy that data collection was carried out in a single city, considering that Brazil is a large and diverse country, which may limit the generalization. Since the WHODAS is an easily applicable, free instrument that is also accessible from mobile phones via the app eWHODAS (https://play.google.com/store/apps/details?id=com.ewhodas_app), prospective future studies could consider this technology in broader cross‐regional studies. Also, we note that convenience sampling tends to select participants who are easily accessible or willing to participate, often from specific areas or contexts, which can lead to bias. However, we believe that the potential sampling bias was mitigated by adopting multiple centers to capture diverse participant profiles. We have also distributed the interviews on different days and times and contacted candidate participants through different medical teams. Finally, despite the validity of the measurement properties of WHODAS for the identified population, we note that almost 30% of the approached participants were excluded for not reaching the acceptance threshold score in the Mini‐Mental State Examination. This can be related to the potential impact of the disease on the mental stress and cognition of the patients, which may impose additional challenges for the application of WHODAS in our target population. However, for clinical proposals, the WHODAS manual presents three modes of administration: by interview, self‐administered, and proxy. This last mode may be administered to family members or a caretaker of the patient who is not able to answer the questionnaire.

## 5 Implications for Physiotherapy Practice

This study contributes valuable insights into WHODAS's measurement properties in the context of breast cancer surgery recovery. We found that the WHODAS 2.0 is a reliable and valid instrument for assessing the functioning of post‐surgery breast cancer patients. The importance of employing valid and reliable instruments to measure their functioning to better design rehabilitation protocols cannot be overstated. Thus, our results will hopefully speed up the adoption of WHODAS as a PROM in clinical trials and be an asset for data interpretation once we provide the SEM and SDC values for WHODAS 2.0, which had not yet been calculated for this population.

We anticipate that our findings can help delineate programs for breast cancer survivors in Brazil, aiming to promote improved quality of life, social participation initiatives, and professional reintegration policies. In addition, clinicians and researchers may use SEM and SDC values as benchmarks to interpret WHODAS scores, both in clinical practice and as part of research projects. Overall, we believe that this study will contribute to a better understanding of the functioning and disability of breast cancer survivors.

## Ethics Statement

The National Ethics Committee approved the project under the registration number CAAE 56060622.0.0000.5050.

## Conflicts of Interest

The authors declare no conflicts of interest.

## Data Availability

The participants of this study did not give written consent for their data to be shared publicly, so due to the sensitive nature of the research, supporting data are not available.

## References

[pri70106-bib-0001] Bray, F. , J. Ferlay , I. Soerjomataram , R. L. Siegel , L. A. Torre , and A. Jemal . 2018. “Global Cancer Statistics 2018: GLOBOCAN Estimates of Incidence and Mortality Worldwide for 36 Cancers in 185 Countries.” CA: A Cancer Journal for Clinicians 68, no. 6: 394–424. 10.3322/caac.21492. 30207593

[pri70106-bib-0002] Brazilian National Cancer Institute (Brasil) . Brazilian National Cancer Institute (Brasil). 2019. https://www.inca.gov.br/sites/ufu.sti.inca.local/files//media/document//a_situacao_do_cancer_de_mama_no_brasil.pdf.

[pri70106-bib-0003] Brazilian National Cancer Institute (Brasil) . Brazilian National Cancer Institute (Brasil). 2022. https://www.inca.gov.br/tipos‐de‐cancer/cancer‐de‐mama.

[pri70106-bib-0004] Brucki, S. M. , R. Nitrini , P. Caramelli , P. H. Bertolucci , and I. H. Okamoto . 2003. “Sugestões para o uso Do mini‐exame Do estado mental no brasil.” Arquivos de Neuro‐Psiquiatria 61, no. 3B: 777–781: (September). 10.1590/s0004-282x2003000500014. 14595482

[pri70106-bib-0005] Castro, S. , and C. Leite . 2017. “Translation and Cross‐Cultural Adaptation of the World Health Organization Disability Assessment Schedule—WHODAS 2.0.” Fisioterapia e Pesquisa 24, no. 4: 385–391. 10.1590/1809-2950/17118724042017.

[pri70106-bib-0006] Castro, S. , C. Leite , C. Osterbrock , et al. 2015. Avaliação de saúde e deficiência: manual do WHO disability assessment schedule. World Health Organization. https://iris.who.int/bitstream/handle/10665/43974/9788562599514_por.pdf.

[pri70106-bib-0007] Coster, S. , K. Poole , and L. Fallowfield . 2001. “The Validation of a Quality of Life Scale to Assess the Impact of Arm Morbidity in Breast Cancer Patients Post‐Operatively.” Breast Cancer Research and Treatment 68, no. 3: 777–781. 10.1023/a:1012278023233. 11727963

[pri70106-bib-0008] Dumas, A. , I. V. Luis , T. Bovagnet , et al. 2019. “Impact of Breast Cancer Treatment on Employment: Results of a Multicenter Prospective Cohort Study (Canto).” Journal of Clinical Oncology 38, no. 7: 734–743. 10.1200/JCO.19.01726. 31834818 PMC7048162

[pri70106-bib-0009] Ferrer, M. , M. Perracini , F. Rebustini , and C. M. Buchalla . 2019. “WHODAS‐BO 2.0: Dados normativos para avaliação de incapacidade em idosos.” Revista de Saúde Pública 53, no. 19: 19. 10.11606/s1518-8787.2019053000586. 30726500 PMC6394378

[pri70106-bib-0010] Gagnier, J. , L. Mokkink , J. Lai , and C. B. Terwee . 2021. “Cosmin Reporting Guideline for Studies on Measurement Properties of Patient‐Reported Outcome Measures.” Quality of Life Research 30, no. 8: 2197–2218. 10.1007/s11136-021-02822-4. 33818733

[pri70106-bib-0011] Jamshidi, F. , M. Farzad , J. Macdermid , A. Varahra , S. A. Hosseini , and M. H. Asgarabad . 2022. “Assessing the Content Based on ICF and Quality Based on Cosmin Criteria of Patient‐Reported Outcome Measures of Functioning in Breast Cancer Survivors: A Systematic Review.” Breast Cancer 29, no. 3: 377–393. 10.1007/s12282-022-01340-6. 35233732

[pri70106-bib-0012] Koo, T. , and M. Li . 2016. “A Guideline of Selecting and Reporting Intraclass Correlation Coefficients for Reliability Research.” Journal of Chiropractic Medicine 15, no. 2: 155–163. 10.1016/j.jcm.2016.02.012. 27330520 PMC4913118

[pri70106-bib-0013] Lourenço, A. , A. Dantas , D. Araújo , et al. 2020. “Prevalência da deficiência e associações clínicas em mulheres sobreviventes ao câncer de mama: Um estudo‐piloto.” Revista Brasileira de Cancerologia 66, no. 2. 10.32635/2176-9745.RBC.2020v66n2.843.

[pri70106-bib-0014] McKinley, R. , T. Manku‐Scott , A. Hastings , D. P. French , and R. Baker . 1997. “Reliability and Validity of a New Measure of Patient Satisfaction With Out of Hours Primary Medical Care in the United Kingdom: Development of a Patient Questionnaire.” BMJ 314, no. 7075: 193–198. 10.1136/bmj.314.7075.193. 9022436 PMC2125677

[pri70106-bib-0015] Mendoza, N. , F. Molero , F. Criado , M. J. Cornellana , and E. González . 2017. “Sexual Health After Breast Cancer: Recommendations From the Spanish Menopause Society, federación espanõla de sociedades de sexología, sociedad espanõla de médicos de atención primaria and sociedad espanõla de oncología médica.” Maturitas 105: 126–131. 10.1016/j.maturitas.2017.02.010. 28268037

[pri70106-bib-0017] Mokkink, L. , C. Terwee , D. Patrick , et al. 2010. “The COSMIN Study Reached International Consensus on Taxonomy, Terminology, and Definitions of Measurement Properties for Health‐Related Patient‐Reported Outcomes.” Journal of Clinical Epidemiology 63, no. 7: 737–745. 10.1016/j.jclinepi.2010.02.006. 20494804

[pri70106-bib-0018] Mokkink, L. B. , C. A. Prinsen , D. L. Patrick , et al. 2019. Cosmin Study Design Checklist for Patient‐Reported Outcome Measurement Instruments. Public Health research institute.

[pri70106-bib-0019] Moro‐Valdezate, D. , S. Peiró , E. Buch‐Villa , et al. 2013. “Evolution of Health‐Related Quality of Life in Breast Cancer Patients During the First Year of Follow‐Up.” Journal of Breast Cancer 16, no. 1: 104–111. 10.4048/jbc.2013.16.1.104. 23593090 PMC3625756

[pri70106-bib-0020] Mukaka, M. M. 2012. “Statistics Corner: A Guide to Appropriate Use of Correlation Coefficient in Medical Research.” Malawi Medical Journal: The Journal of Medical Association of Malawi 24, no. 3: 69–71. 23638278 PMC3576830

[pri70106-bib-0029] Oliveira, I. S. , L. C. M. Costa , A. C. T. Manzoni , and C. M. N. Cabral . 2014. “Assessment of the Measurement Properties of Quality of life Questionnaires in Brazilian Women with Breast Cancer.” Brazilian Journal of Physical Therapy 18, no. 4: 372–383. 10.1590/bjpt-rbf.2014.0045. 25075998 PMC4183258

[pri70106-bib-0021] Ovando, A. , C. DallAgnol , L. J. Merlyn , R. Andrade Momo , and S. S. De Castro . 2024. “The Brazilian Version of the World Health Organization Disability Assessment Schedule (WHODAS 2.0) Is Reliable and Valid for Chronic Stroke Survivors.” Topics in Stroke Rehabilitation 31, no. 2: 211–220. 10.1080/10749357.2023.2207293. 37120851

[pri70106-bib-0022] Polit, D. 2014. “Getting Serious About Test‐Retest Reliability: A Critique of Retest Research and Some Recommendations.” Quality of Life Research 23, no. 6: 1713–1720. 10.1007/s11136-014-0632-9. 24504622

[pri70106-bib-0023] Schmidt, M. , S. Scherer , J. Wiskemann , and K. Steindorf . 2019. “Return to Work After Breast Cancer: The Role of Treatment‐Related Side Effects and Potential Impact on Quality of Life.” European Journal of Cancer Care 28, no. 4: e13051. 10.1111/ecc.13051. 31033073

[pri70106-bib-0024] Silva, C. , I. Coleta , A. Amaro , J. Alvarelhao , A. Queiros , and N. Rocha . 2013. “Adaptation and Validation of WHODAS 2.0 in Patients With Musculoskeletal Pain.” Revista de Saúde Pública 47, no. 4: 752–758. 10.1590/s0034-8910.2013047004374. 24346666

[pri70106-bib-0025] Tavakol, M. , and R. Dennick . 2011. “Making Sense of Cronbach’s Alpha.” International Journal of Medical Education 2: 53–55. 10.5116/ijme.4dfb.8dfd. 28029643 PMC4205511

[pri70106-bib-0026] Ustun, T. , N. Kostanjsek , S. Chatterji , et al. 2010. Measuring Health and Disability: Manual for Who Disability Assessment Schedule (WHODAS 2.0). World Health Organization. https://www.who.int/publications/i/item/measuring‐health‐and‐disability‐manual‐for‐who‐disability‐assessment‐schedule‐(‐whodas‐2.0.

[pri70106-bib-0027] World Health Organization 2013. Como usar a CIF: Um manual prático para o uso da classificação internacional de funcionalidade, incapacidade e saúde (CIF).

[pri70106-bib-0028] Zhao, H. , Y. Liu , H. Li , L. Ma , Y. J. Zhang , and J. Wang . 2013. “Activity Limitation and Participation Restrictions of Breast Cancer Patients Receiving Chemotherapy: Psychometric Properties and Validation of the Chinese Version of the WHODAS 2.0.” Quality of Life Research 22, no. 4: 897–906. 10.1007/s11136-012-0212-9. 22684528

